# 胸腔镜手术下肺小结节常见定位方法研究进展

**DOI:** 10.3779/j.issn.1009-3419.2018.08.09

**Published:** 2018-08-20

**Authors:** 诚 沈, 鹏飞 李, 珏 李, 国卫 车

**Affiliations:** 610041 成都，四川大学华西医院胸外科 Department of Thoracic Surgery, West China Hospital, Sichuan University, Chengdu 610041, China

**Keywords:** 肺小结节, 电视胸腔镜手术, 定位, Solitary pulmonary nodule, Video-assisted thoracoscopic surgery, Localization

## Abstract

最近，伴随着高分辨多层电子计算机断层扫描（computed tomography, CT）的大量普及，肺小结节的诊断也日益增多，尤其是在伴有肺癌高危因素的患者行CT筛查时尤为明显。电视辅助胸腔镜手术对于肺小结节的诊断和治疗提供了一种全新的微创治疗方式，胸腔镜手术后给患者带来的疼痛感减少、住院时间缩短、手术并发症减少等特点，使其推广更为广泛。如何精准定位及标记病灶，以助电视胸腔镜下切除病灶的方法层出不穷。本文综述近年来胸腔镜下肺小结节定位的各种技术手段，并对各种方法的利弊进行总结及分析。

## Abstract (en)

Recently, with the proliferation of high-resolution computed tomography (CT), the frequency of small pulmonary nodules appears higher and more precise than previously estimated, especially in CT screening in patients with high risk factors for lung cancer. Video-assisted thoracoscopic surgery (VATS) provides a new minimally invasive treatment for the diagnosis and treatment of small pulmonary nodules. The VATS results in less pain, shorter hospital stay, and reduced surgical complications, making it more widely available. How to accurately locate and mark lesions is important for video-assisted thoracoscopic surgery. This article reviews the various techniques used to locate pulmonary nodules in surgery in recent years and summarizes the advantages and disadvantages of them.

最近，伴随着高分辨多层电子计算机断层扫描技术（high resolution computed tomography, CT）的大量普及，肺小结节（small pulmonary nodules, SPNs）的诊断也日益增多。尤其是在伴有肺癌高危因素的患者行CT筛查时尤为明显。对于肺小结节的定义是指边界清楚、类圆形或不规则形病灶，影像学表现为密度增高的影像、直径≤30 mm、周围可被含气肺组织包绕^[1, 2]^。相关文献^[3]^报道，肺结节的恶性概率跟结节的大小、密度等因素相关，直径 < 5 mm的极微小结节，恶性的可能性为0%-1%；直径为5 mm-10 mm微小结节时，恶性的可能性6%-28%；当结节直径 > 20 mm时，恶性可能性为64%-82%。在临床工作中，病理学诊断依旧是明确小结节性质的“金标准”。

随着胸外科的发展，电视辅助胸腔镜手术（video-assisted thoracoscopic surgery, VATS）为肺小结节的诊断和治疗提供了一种全新的微创治疗方式，胸腔镜手术后给患者带来的疼痛感减少、住院时间缩短、手术并发症减少等特点，使其推广更为广泛^[4, 5]^。然而，胸腔镜手术对于部分肺小结节的处理依然能力有限，大多数直径 < 1 cm或胸膜下深度超过5 mm的小结节，术中无法通过视觉、手指触诊、腔镜器械滑行进行定位，使得手术本身的操作时间延长，尤其是对于本身质地较软的纯磨玻璃样结节（ground-glass opacity, GGO），更增加了手术难度^[6, 7]^。因此，如何在术前准确定位病灶，以帮助术者在术中顺利寻找病灶，实现准确定位，减少中转开胸率，依然是胸外科医生需要解决的问题之一。目前，胸腔镜下肺小结节的定位方法大体上可分为注入液体材料介导定位、经皮置入固态材料介导定位、影像介导定位3类方法。本文综述近年来胸腔镜下肺小结节定位的各种技术手段，并对各种方法的利弊进行总结及分析。

## 1 液体材料介导定位

### 1.1 亚甲蓝定位法

Lenglinger等^[8]^最早提出了可通过使用亚甲蓝染色的方法进行体内结节的定位。由于亚甲蓝材料容易获取，价格实惠以及在临床操作过程中疼痛相对较轻，使得其在临床应用中得以实施^[9]^。Stephenson及其团队研究者们^[10]^通过2年收集了30例用此方法标记的患者资料总结发现，结节平均大小在8 mm，结节距离肺表面深度平均为17 mm，所有结节均可以在胸膜表面看到染料的着色区域。术后无一例患者出现定位相关并发症。Findik等^[4]^收集了从2013年4月-2016年10月期间，共11例实施定位的患者资料进行临床评价。所有的这些患者均进行了^18^F-FDG PET-CT扫描检查。术前2 h将患者在CT引导下美蓝染色。最终发现，11例患者的结节平均大小为8.7 mm，距离肺表面平均深度为12.7 mm。有4例患者结节位于左肺（2例位于左肺上叶，2例位于左肺下叶）；另外的7例患者中有4例位于右肺下叶，2例位于右肺上叶，1例在右肺中叶。术后患者也均未发生定位相关的并发症。

然而，在实际操作中，亚甲蓝依然存在一些不足，一方面，亚甲蓝弥散速度快，常常要求临床医生在注射定位后的3 h内进行胸腔镜手术，这对于手术安排和衔接存在一定影响。另一方面，其容易扩散和被干扰的特点，使得肺表面定位区域变大，造成肺切除范围扩大。Jin等^[11]^通过比较单纯亚甲蓝注射和亚甲蓝联合碘油的混合液（5:1）在定位中的作用结果发现，动物实验中混合液的染色范围较单纯亚甲蓝范围小（0.6 cm *vs* 1 cm, *P* < 0.001），而混合液的染色效果明显比单纯亚甲蓝染色能力强（*P*=0.010），混合液染色评分为优秀的病例占17例（81%）和而仅有4例（19%）亚甲基蓝染色的病例评分为优秀（*P*=0.011）。研究结果也进一步亚甲蓝和碘油的混合液在肺小结节的定位中有其重要意义。Kleedehn等^[12]^还比较了CT引导下亚甲蓝注射定位与Hook-wire定位的区别。109例患者中，52例实行了带勾金属丝Hook-wire定位，另外57例进行了亚甲蓝染色定位。术后的并发症比较中，Hook-wire定位组比亚甲蓝定位组出现的多，但最终数据结果没有统计学意义，值得注意的是，Hook-wire定位组中出现了7例金属丝脱落的情况，而亚甲蓝染色组均定位准确。

### 1.2 碘油、含碘对比剂定位法

碘油是一种价格便宜和易于获取的造影剂，常用于消化道的造影。其在注入体内后，能比周围组织吸收更多的X线，使得显影更为满意，同时其在体内比钡剂排泄快，停留时间短。Mogi等^[13]^研究者通过收集了自2007年-2014年，7年间56例CT引导下碘油造影肺小结节定位的患者临床资料，结果发现，所有患者均在术前成功得到定位，结节的大小3 mm-20 mm（10.5±4.35）mm，定位针从皮肤到结节的距离3 cm-12 cm（5.08±1.55）cm。在术后并发症发生方面，发生气胸的患者21例（37.5%），只有1例患者需要暂时性胸腔闭式引流。肺实质内出血的发生患者为9例（16.1%），但严密观察患者病情变化后并无1例产生症状加重的患者。56例患者中有55例（98.2%）成功接受了胸腔镜肺切除术。有1例由于碘油造影剂在术中未能显影良好，病人遂转为开胸手术。碘油弥散速度较亚甲蓝慢，显影区域表现稳定，定位精确度高，同时肺内存留时间较长，在定位室与手术室间的衔接时间相对宽裕；此外，相比于钡剂造影，其在患者最终的病灶诊断结果上不会产生干扰^[14-16]^。在最新的研究中，Kim等^[17]^提出将印度墨水（India ink）与碘油相结合的方式，进行肺小结节的定位。印度墨水在胃肠道手术中的定位取得了一定的满意结果^[18, 19]^，然而在肺内小结节中却未尝试。基于其显色较亚甲蓝染色更深，胸腔镜下观察更为典型的特点，Kim的团队成功为1例患者实施定位并顺利实行了手术。尽管碘油造影有很多优点，然而，临床应用过程中依然存在一些不足，可能会存在肺动脉栓塞的风险^[20]^；部分报道提出易导致定位组织炎性反应，甚至发生肺炎^[21]^。由于碘油定位需手术医师在X线透视下操作，这也限制了碘剂定位在临床中的使用。此外，还包括甲状腺功能亢进、甲状腺肿瘤、有严重心、肝、肺疾患、急性支气管炎症和发热患者也要慎用。

### 1.3 医用胶定位法

医用胶在VATS手术中的定位方法开展的时间并不长，其本质是以α-氰基丙烯酸正辛酯为主体胶，添加聚甲基丙烯酸甲酯而成。医用胶遇到微量阴离子物质，如人体内的血液、体液或组织液时，在室温下即迅速发生聚合反应，固化成膜并与接触的组织表面紧密镶嵌。医用胶最大的特点就是无毒无害，生物安全性好。国外的文献少有报道，国内的几项研究^[22-25]^反映出了其临床应用中的巨大优势：①医用胶形成的硬节在胸腔镜下用手指或者器械都可以轻易触及，具有良好的术中辨识度。②硬结不易脱落，定位牢固可靠。③并发症发生率更低。医用胶的固化过程本身就可封闭胸膜或血管的破口，起到防止气胸及止血的作用。④医用胶固化后短时间内不会被组织吸收，更长的衔接期有利于患者在定位室与手术室之间的协调安排。然而，临床中的实际运用还是存在一些问题。因为这种胶水是工业合成的，也具有一定的刺激性气味，如果注射速度过快，该气味可能随着患者的呼吸进入气管造成刺激性咳嗽，同时，穿刺定位所带来的胸痛和气胸也时有发生。

## 2 金属材料介导定位

### 2.1 带钩金属丝定位法（Hook-wire）

Hook-wire定位是最早的也是应用最为广泛的结节定位法^[26]^，其操作的原理就是一根前端成钩状弯曲的细长金属丝，在低剂量薄层CT扫描首次定位后，通过套针经皮穿刺进入到胸腔到达病灶或其周围后，重复局部低剂量断层扫描，将套筒针尖斜面朝向病灶方向，释放金属丝并回收套针，金属丝前段钩状展开，固定在结节周围，这时轻拉金属线会有阻力感，再次局部低剂量断层扫描，确定金属钩锚定膨胀良好并锚定病灶后，剪断金属丝留在体外部分，最后转至手术室行VATS手术。

Xu等^[27]^研究161例病灶结节 < 20 mm的患者均通过Hook-wire定位系统实行了术前的定位，共定位结节168个。从临床特点、影像学表现以及术后的病理结果进行了综合的评估和分析发现，71个（42.3%）病灶影像学表现为纯磨玻璃样结节影（pure ground-glass opacities, GGOs），59个病灶（35.1%）影像考虑为GGO伴部分实性成分，38个结节（22.6%）是实体病变。在病理学诊断结果上，纯GGO中有49.3%诊断为非典型腺瘤样增生（atypical adenomatous hyperplasia, AAH），GGO伴部分实性成分者中有54.2%为肺癌，65.8%的实性结节为良性病变。在168个病灶安置过程中，成功安置164例（97.6%）结节，安置失败的4例病灶均位于胸膜下的结节，由于反复穿刺致肺组织破损至出血，Hook-wire难以固定而滑脱。在161例患者中，只有3例患者（1.9%）主诉疼痛不适，然而他们的疼痛评分均 < 2分。定位点局部出血的患者有2例（1.2%），均无需处理。低血压（由于血管迷走神经反应引起的）患者有2例（1.2%），二者均在予以地塞米松处理和处于水平位置得到迅速的缓解。由于该方法定位操作过程较为成熟，操作内容相对简单且操作时间耗时短，穿刺针定位点明确，方便病灶的切除^[28]^，同时可以满足手术医师在胸腔镜直视下观察，而无需X线透视定位，因此在临床上得以广泛应用，类似的结果，均在多篇文献中得到证实^[29-31]^。然而，金属丝易移位甚至脱落的特点依然是Hook-wire定位失败的主要原因。有文献报道，若插入距离距胸膜表面 < 5 mm，将增加移位的风险，同时，定位后未限制患者活动，并由于手术室周转问题未能尽快转送患者进行手术也是造成移位和脱落的高危因素。Chen等^[32]^报道了1例考虑为右肺上叶小结节的患者，通过Hook-wire定位后进行VATS手术。术中发现定位金属丝既不在胸腔内也不在肺内，最终通过床旁胸片提示盘曲的金属丝脱落到了胸壁。其次，定位后易出现气胸、出血和疼痛等，也是常见的定位后并发症^[33, 34]^。因此，针对这些问题可考虑行Hook-wire定位联合亚甲蓝染色起到双重定位作用，提高了定位成功率。

### 2.2 微弹簧圈定位法

微弹簧圈最早是用于血管内的栓塞。1992年就有个案报道关于使用微弹簧圈应用于肺结节的定位，但完全是处于初步探索阶段^[35]^。微弹簧圈的使用流程是先将套管针将微弹簧圈的前半段经皮穿刺后在肺内结节周围释放定位，随后将套管针退至脏层胸膜外，释放微弹簧圈的余下部分，这样术中可以看到脏层胸膜表面的线圈，定位穿刺点，形成哑铃形的线圈。手术过程中通过探查位于脏层胸膜外的线圈大致位置，根据弹簧圈头端与病灶的位置关系及病灶深度，对肺部小结节进行较准确的定位^[36]^。一项前瞻性随机对照实验中，术前病灶直径≤15 mm肺结节被随机安排定位组和非定位组。其总共收集了2010年-2012年间56例患者的病历资料。其中，29例在微弹簧圈定位组，而有27例在非定位组。两组的基线资料是基本相似的（包括年龄、性别、第1秒用力呼气容积以及结节的大小和所在深度）。结果发现，微弹簧圈定位组较非定位组在肺楔形切除术中成功率更高（27/13, *P* < 0.001）且在缩短手术时间上也有明显改善[(37±39) min *vs* (100±67) min, *P* < 0.001]。这项研究肯定了微弹簧圈在临床肺结节定位中的重要价值。随后，有多为学者从不同的角度研究了微弹簧圈在肺手术中的应用意义，进一步强调了其高效、准确、微创切除病灶并有效缩短手术及住院时间的优点^[37-39]^。在最新的一项临床研究中提到，在回顾性分析了192例患者共有206个结节的微弹簧圈定位中，微弹簧圈脱位者有6例（2.9%），微弹簧圈定位成功率为97.1%。通过*Logistic*回归分析发现微弹簧圈针头的定位深度和气胸发生是微弹簧圈定位的独立影响因素^[40]^。然而，其材料昂贵以及类似Hook-wire的脱落情况，依然是临床需要解决的问题。

在2017年Park等^[41]^发表的一篇系统回顾和荟萃分析比较了3种肺结节定位方法在VATS中的成功率和并发症发生率：包括带钩金属丝定位、微弹簧圈定位和碘油定位。结果共纳入46项临床研究。Hook-wire、微弹簧圈和碘油定位的成功率分别为0.98（95%CI: 0.97-0.99），0.98（95%CI: 0.96-0.99）和0.99（95%CI: 0.98-1.00），相应的手术靶区成功率分别为0.94（95%CI: 0.91-0.96），0.97（95%CI: 0.95-0.98）和0.99（95%CI: 0.98-1.00）。此外，VATS成功率分别为0.96（95%CI: 0.94-0.97），0.97（95%CI: 0.94-0.99）和0.99（95%CI: 0.98-1.00）。在并发症发生方面，三者的气胸发生率分别为0.35（95%CI: 0.28-0.43）、0.16（95%CI: 0.07-0.34）和0.31（95%CI: 0.20-0.46），出血率分别为0.16（95%CI: 0.11-0.23）、0.06（95%CI: 0.03-0.11）和0.12（95%CI: 0.05-0.23）。总结发现，Hook-wire定位的手术靶区成功率相对较低。碘化油定位的总体成功率最高，微弹簧圈定位产生最低的并发症发生率。

## 3 影像介导定位

常见的影像介导下的定位包括术中超声定位、术中近红外线扫描定位和计算机导航定位。

随着技术手段的革新，越来越多的学者报道了超声定位在肺手术中的应用。在过去，超声检查对于肺部疾病的诊断存在很大的局限性，究其原因是因为肺组织是一个充满气体的脏器，人体软组织与气体的声音阻抗差别极大，声束难以穿透肺组织，而在脏层胸膜形成近似全反射的强回声，与X线和CT等放射学检查相比，这是超声检查在肺部疾病应用中最大的障碍。早在1992年，就有报道关于应用超声引导下肺部小结节的穿刺活检^[42]^。近年来，随着计算机技术、超声探头及显示器的改进，高分辨率超声在肺部结节良恶性病变鉴别诊断中的作用日益突出。作为一种安全、经济、无创的方法，多篇文献已报道了其在术中对微小结节或GGO的成功应用^[43, 44]^。但是超声对操作者的依赖性较高，同时更多要求在患肺完全塌陷时进行，而对于慢性阻塞性肺疾病的患者中实施有一定困难。

在2015年的一篇文献报道^[45]^中，提到了使用近红外成像（near-infrared imaging, NIR）的影像识别技术。原理就是患者在术前注射荧光显色剂（吲哚青绿），术中用NIR胸腔镜探查时可以通过比对荧光区域与术前胸部CT扫描定位点位置相符，并通过识别荧光区域的边缘进行肺小结节的切除。但该技术的弊端在于可能存在假阳性的荧光显像。

计算机辅助手术技术（image-guided surgery, IGS）极大的促进了微创手术的发展。其操作原理是将患者CT资料导入导航仪计算机，计算机进行图象重建与穿刺路线模拟，根据体位将患者胸部的三维空间坐标体系与CT图像的三维坐标体系的重叠，以确定穿刺针的进针方向和深度^[46, 47]^。Moghissi等^[48]^发表的综述中提到，采用该技术能清晰显示病灶与支气管、血管和胸膜的空间解剖关系，弥补原始横断层图像的不足，使医生明确手术器械相对患者解剖结构的具体位置，给外科手术带来很大便利。然而，其具体实施过程中需要专门的设备和仪器，限制了其在临床的广泛开展。

## 4 其他技术

### 4.1 电磁导航支气管镜引导技术

电磁导航支气管镜（electromagnetic navigation bronchoscopy, ENB）是近年来出现的一项新微创诊断技术。利用ENB引导标记孤立性肺结节，已被广泛应用于VATS术前定位^[49, 50]^。ENB引导放置螺旋弹簧基准标记具有更好的保留率，与线性基准标记相比具有较低的并发症发生率^[51]^。其次，在2016年的一项研究^[6]^中也提出ENB引导胸膜染料标记在肺小结节的定位中是安全可行的。Sun等^[52]^首次报道了1例ENB导航下联合Hook-wire进行肺小结节的亚甲蓝染色定位，充分说明此方法的实用性。

### 4.2 放射性同位素

示踪剂定位放射性核素具有较长的半衰期，同时可通过探测仪示踪确定放射性核素的区域。借助其自身的特点，临床上可在CT引导下将放射性核素粒子在经皮穿刺注射到肺内病灶周围进行定位。Galetta等^[53]^收集了2007年11月-2013年5月，共有112例患者接受术前放射性示踪剂注射，所有患者均获得成功标记。总体而言，共有123个肺结节进行了定位和切除。平均定位结节大小为9 mm，结节距胸膜的平均距离为12 mm。70例（62.5%）患者进行了胸腔镜肺切除术，36例（32.1%）开胸手术，6例（5.4%）胸腔镜中转开胸手术。由于可以连续使用探针来定位肺结节，此方法实现了100%的成功率。这种方法对于较表浅的病灶定位较准确，但设备要求特殊，价格昂贵，且存在辐射危害，外科医生和放射科医师可能暴露于辐射之中，且对于较深的肺结节定位模糊，限制其在临床上的使用。

总之，肺小结节的每一种定位方法都有它的优点和缺点。在缺乏完整多中心的临床研究实验的结果情况下，暂不可能建立起标准的肺小结节定位的金标准。此外，医生也可以基于其定位设备的限制而选择不同的技术。当前最为理想的定位技术应当是高精准度，定位后并发症发生率较低，价格低廉，对人体安全无害且可以广泛使用。相信随着医学的发展，更多对患者有利、对手术者方便的定位方法将会出现，所以仍需不断探索。
